# Human and Mouse CD137 Have Predominantly Different Binding CRDs to Their Respective Ligands

**DOI:** 10.1371/journal.pone.0086337

**Published:** 2014-01-21

**Authors:** Ling Yi, Yanlin Zhao, Xiaojue Wang, Min Dai, Karl Erik Hellström, Ingegerd Hellström, Hongtao Zhang

**Affiliations:** 1 Department of Central Laboratory, Beijing Chest Hospital, Capital Medical University and Beijing Tuberculosis and Thoracic Tumor Research Institute, Beijing, People's Republic of China; 2 Chinese Center for Disease Control and Prevention, Beijing, People's Republic of China; 3 Department of Pathology, Harborview Medical Center, University of Washington, Seattle, Washington, United States of America; National Cancer Institute, NIH, United States of America

## Abstract

Monoclonal antibodies (mAbs) to CD137 (a.k.a. 4-1BB) have anti-tumor efficacy in several animal models and have entered clinical trials in patients with advanced cancer. Importantly, anti-CD137 mAbs can also ameliorate autoimmunity in preclinical models. As an approach to better understand the action of agonistic and antagonistic anti-CD137 mAbs we have mapped the binding region of the CD137 ligand (CD137L) to human and mouse CD137. By investigating the binding of CD137L to cysteine rich domain II (CRDII )and CRDIII of CD137, we found that the binding interface was limited and differed between the two species in that mouse CD137L mainly combined with CRDII and human CD137L mainly combined with CRDIII.

## Introduction

CD137 belongs to the third group of receptors encoded by the TNF superfamily which also includes OX40, CD27, CD30, and CD40. These receptors are all costimulatory molecules and act at different stages in T and B cell activation to modulate the immune response [1]–[3]. CD137 is expressed by multiple myeloid cells including activated effector CD8^+^ and CD4^+^ T cells, natural killer (NK) cells, NK/T cells, dendritic cells (DCs), macrophages, neutrophils, eosinophils [4], and according to recent data, also by regulatory T cells (Tregs), activated B cells, mast cells and endothelial cells in tumor capillaries [4]–[7]. Engagement of CD137 boosts proliferation of T cells, activates their effector functions, survival and establishes immunological memory [8]. CD137 signaling promotes a T cell response by activating PI-3-kinase and Akt/PKB signaling pathway, increases expression of Bcl-XL and Bfl-1 and enhances IFN-γ secretion to polarize Th1 differentiation [9]. CD137-deficient mice have a decreased CD8^+^ T-cell response to virus infection [10]. Baessler and colleagues recently reported that the engagement of CD137 on mouse and human NK cells had opposite effects in that CD137 functions as an inhibitory receptor in humans and as a stimulatory receptor in mice [11].

Administration of anti-CD137 mAbs has significant therapeutic activity against established tumors in several mouse models, including tumors that are poorly immunogenic [12]–[14]. Engagement of CD137 can also down-regulate immune responses for therapeutic benefit in a variety of mouse models of autoimmune diseases [15]–[17]. Two fully human anti-CD137 mAbs have been developed and entered phase I–II studies in patients with advanced solid tumors or B-cell malignancies [18]–[19]. However, there is a concern using these mAbs in view of the expression of CD137 and its ligand by a variety of normal cells as well as the fact that opposite biological effects and severe side-effects have been observed [20]–[21].

Antibodies to costimulatory receptors can be either antagonistic or agonistic. There are similarities between the toxicities induced by engaging CD137 and CD28, including a systemic inflammatory response involving CD4^+^ T cells and a “cytokine storm” [22]. For example, two different mAbs to CD28, JJ316 and JJ319 trigger different functional signals via CD28 with JJ316 being a hypercostimulatory activating mAb [23]. The mechanisms responsible for the different between different mAbs to the same costimulatory molecule are not known.

Little is known about the molecular interactions that are responsible for the binding of CD137 to CD137L. Predicting specific interactions on the basis of structural information alone has not been possible. Data from multiple mutagenesis and binding studies have allowed the identification of amino acid residues in the extracellular domain of TNF which are critical for receptor binding [24]–[28]. The binding between CD40 and CD40L and between OX40 and OX40L [29]–[30] has been determined. In contrast, no crystals of CD137-CD137L have been produced [31]. In this report we have mapped the mouse and human CD137 regions which are responsible for binding to the corresponding natural ligands and we analyze their structures.

## Materials and Methods

### Ethics statement

Research involves taking peripheral blood from healthy human and spleen from mice, and all these had been approved by Beijing Tuberculosis and Thoracic Tumor Research Institute Ethics Committee. Animal experiments have been conducted according to relevant national and international guidelines. All participants provided written informed consent prior to participation in the study.

### Isolation, activation of lymphocytes and cDNA preparation

Human peripheral blood mononuclear cells (PBMCs) from healthy donors were isolated by Ficoll-Hypaque gradient centrifugation, resuspended at 1×10^6^/ml in RPMI 1640 medium (Gibco, Carlsbad, CA, USA) supplemented with 10% fetal bovine serum (Gibco) and activated by incubation with phytohemagglutinin (PHA, Sigma, St Louis, MO, USA) at 50 µg/ml for 36 h at 37°C. Lymphocytes from mouse spleens were prepared after lysing the erythrocytes with ammonium chloride and activated by incubation with concanavalin A (ConA,Sigma) at 5 µg/ml for 36 h at 37°C in 10% FBS RPMI 1640 medium. Expression of CD137 on the T cells was confirmed by flow cytometry (FACS Caliber, BD, San Jose, CA, USA) after double staining with FITC-conjugated anti-CD3 (OKT3, ebioscience, San Diego, CA, USA) and PE-conjugated anti-CD137(BD Biosciences, San Diego, CA, USA). RNA was extracted from activated and unstimulated human and mouse lymphocytes using TRIzol (Life Technologies, Carlsbad,CA, USA), and single-stranded cDNAs of IgG-Fc, CD137 and CD137L were synthesized using poly(dT) primers and M-MLV RT (Promega Corporation, Madison,WI, USA).

### Construction of expression vectors

Genes encoding human IgG1 and mouse IgG2a heavy chain Fc fragments were inserted, respectively, into pcDNA3.1 vector (Life Technologies, Carlsbad, CA, USA) at its multiple cloning sites to construct different Fc-fusion protein expression vectors. The published sequences of human IgG1-Fc and mouse IgG2a-Fc were acquired [32]–[33]. The gene encoding human IgG1 (hinge-CH2-CH3) was obtained by PCR using the following primers: sense 5′-CGCAAGCTTTAAGGATCCATCTGCAACGTGAATCAC-3′ and antisense 5′-TGCGAATTCTTACGTCGCACTCATTTACCCGGAGAC -3. The amplified DNA fragment in a sequence order of Hind III+BamHI+ human IgG1 (hinge-CH2-CH3)+ XbaI was first cloned into T-easy vector (Promega Corporation, Madison,WI, USA) and subcloned into pcDNA3.1 vector by HindIII and XbaI digestion. This vector, pCDH, was used to express CRDs of the CD137 extracellular region with its own leader sequence. DNA sequence of “HindIII+HE4 leader+AgeI+human IgG1(hinge-CH2-CH3)+XbaI” and “HindIII+HE4 leader+AgeI+mouse IgG2a(hinge-CH2-CH3)+XbaI” were synthesized by Sangon Biotech (Shanghai, China) and then subcloned into pcDNA3.1 vector to construct two other expression vectors, pCDH-L and pCDM-L, both containing a HE-4 (human epididymis protein 4) leader sequence. These two vectors were used for expressing the genes of CD137L extracellular region and truncated CD137 CRDs.

### Production of CD137 CRDs and CD137 Ligand fusion proteins

The designed fragments of truncated CD137 and its ligand were amplified by PCR from cDNA and subcloned into the pCDH, pCDH-L and pCDM-L vectors. The primers used for amplification of various mouse and human CRDs or CD137L are listed in Table S1. A pair of primers: m1-AAG AGG ACA CGA AGG AGC TGG TGG TCG CCA and m2-TGG CGA CCA CCA GCT CCT TCG TGT CCT CTT, was used to perform a site directed mutagenesis (g→c) at the 405^th^ base of human 4-1BBL extracellular domain to destroy the AgeI restriction enzyme site through overlap PCR without any 4-1BBL amino acid changed [2], and then human 4-1BBL extracellular domain was cloned into pCDH-L and pCDM-L by using AgeI+XbaI restriction site. The point mutation was verified by sequencing. All the constructed expression plasmids were transfected into COS-7 cells with lipofectamine 2000 (Life Technologies, Carlsbad, CA, USA) to produce human and mouse IgG fusion proteins, which were subsequently purified with rProtein A Sepharose (GE healthcare, Uppsala, Sweden ) and eluted by glycine elution buffer. The mouse CD137 extracellular domain with hFc tail (mE-hFc) was obtained by transfection of COS-7 cells using DEAE dextran (Sigma, St Louis, MO, USA).

### Quantitation of expressed proteins

After purification by affinity chromatography, the expressed fusion proteins were quantitated by spectrophotometer analysis, and a sandwich ELISA was established to measure the concentration of human CD137. In brief, anti-human CD137 mAb h4-1BB-M127 (BD Pharmingen, San Diego, CA, USA) was used as a capture antibody, and a serially diluted solution of hCD137-hFc fusion proteins (Sino Biological Inc, 10041-H03H, China) with a purity over 95% was used as reference standard. This was followed by addition of a biotin-labeled anti-human CD137 mAb (BD Pharmingen, San Diego, CA, USA), 4B4-1, which recognizes a different epitope after which strep-HRP was added followed by TMB (Sigma, St Louis, MO, USA) substrate solution as color developing agent, and OD450 nm was determined. By establishing standard curves, the concentration of transfected culture supernatants of hCD137-hFc fusion protein was determined.

### ELISA assays for CD137 and CD137 Ligand binding

For mouse CD137 and CD137L binding experiments, microtiter plates (Nunc, Roskilde, Denmark) were coated overnight with purified mCD137L-mFc (mouse CD137L extracellular domain with mouse IgG-Fc fusion protein) at 1 µg/ml, blocked by 5% milk (OXID LTD, Basingstoke, Hampshire, England) for 1 h at 37°C after which expression supernatants of mE, mE2, mE3, mE4(mouse CD137 CRDs with human IgG-Fc fusion proteins) were added for 2 h at 37°C. For detection, microtiter plates were incubated with goat anti-human IgG-HRP antibodies (Jackson Immuno Research laboratories Inc, PA, USA) at 37°C for 1 h. In subsequent quantitaion ELISA experiment, using the same procedures, expression supernatants were replaced by purified mE, mE2, mE3, mE4 proteins, concentrations ranging from 0.1 µg/ml to 10 µg/ml. TMB (Sigma, St Louis, MO, USA) substrate solution was used to detect positive reactions which were evaluated at OD450 nm.

To examine the ability of human CD137 and various CRDs binding to human CD137L, microtiter plates were coated with purified hCD137L-mFc (human CD137L extracellular domain and mouse IgG-Fc fusion protein) at 1 µg/ml, after which expression supernatants of hE, hE2, hE3, hE4 (human CD137 CRDs with human IgG-Fc fusion proteins) were added, followed by goat anti-human IgG-HRP. Parallel to this, microtiter plates were coated with purified hCD137L-hFc (human CD137L extracellular domain with human IgG-Fc fusion protein) which was followed by adding expression supernatant of hE300, hE3003, hE3004 (human CD137CRDs with mouse IgG-Fc fusion proteins) and detected by goat anti-mouse IgG-HRP. Experiments were also done in which microtiter plates were coated with purified hCD137L-mFc, 1 µg/ml, after which a range of concentrations of purified hE3 (CRDI and CRDII) from 0.2 µg/ml to 10 µg/ml was added to further confirm the binding of recombinant hE3 to hCD137L.

For cross-binding experiments, microtiter plates were coated with purified hCD137L-mFc and mCD137L-mFc at 1 µg/ml, after which expression supernatants of hE and mE were added. In other experiments, expression supernatants of hE, hE4 and mE, mE3, mE4 were added for cross binding of human CD137L to mouse CD137 CRDs. P/N (average OD value of positive wells/average OD value of negative wells ) >2.1 was chosen as a positive ELISA reaction. The error bars represent standard deviation of the means based on three replicates of one representative experiment.

### Monoclonal antibodies and blocking experiments

Rat hybridoma 2A [34], which is specific for mouse CD137, was kindly provided by Dr. Lieping Chen from Yale University. Another anti-mouse CD137 hybridoma, D4-67, was established in our laboratory by immunizing a Lewis rat by i.p. and s.c. injection of 100 µg/0.3 ml of mCD137-hFc fusion protein mixed with Freund's Complete Adjuvant (Sigma, St Louis, MO, USA) followed by 3 subsequent injections i.p. and s.c. of 100 µg/0.3 ml of mCD137-hFc given every second week together with Freund's Incomplete Adjuvant (Sigma). One month after the last immunization, the rat was boosted by i.p. injection and euthanized 4 days later when spleen cells were hybridized with mouse P3-X63-AG8.653 myeloma cells using standard procedures. Wells containing hybridoma cells were screened for binding to mCD137-hFc fusion protein and not to mCD28-hFc fusion protein. D4-67 was cloned from a high producing well.

Purified hCD137-hFc at a concentration of 1.0 µg/ml and mouse anti-human CD137mAbs and OKT3 at a concentration of 5 µg/ml or 25 µg/ml were mixed and incubated at 4°C overnight. Microtiter plates were coated with purified hCD137L-mFc at 1 µg/ml and incubated with blocking solution, after which the above mixture was added and incubated for 2 h 37°C followed by mouse anti-human Fc-HRP for detection. Two different anti-human CD137 mAbs (4B4-1 and h4-1BB-M127, BD Pharmingen) were serially diluted from 0.1 mg/ml to 10 µg/ml and used for similar ELISA blocking experiments. mCD137-hFc at a concentration of 1 µg/ml was mixed with two rat anti-mouse CD137mAbs (2A and D4-67) and added to mCD137L-mFc coated plates, which was followed by mouse anti-human-Fc-HRP for detection.

### PAGE and Western blot analysis

Expressed proteins were purified with rProtein A, mixed with loading buffer, boiled for 5 minutes, separated on a 10% SDS polyacrylamide gel and transferred onto a nitrocellulose membrane (PALL Corporation, Pensacola, FL, USA). The membrane was stained with Ponceau S at RT, washed with water until the background was white. The membrane was incubated in 5% milk-PBS for blocking, subsequently goat anti-mouse IgG-HRP or goat anti-human IgG-HRP were added. Chemiluminescent substrate for HRP (Thermo scientific, Rockford,IL, USA) was used to visualize the protein band. In addition, CD137-Fc and CD137L-Fc, in loading buffer without 2-ME and without being boiled, were run on PAGE gel after which the gel was stained with Coomassie Blue Stain Solution (Amesico, Solon, OH 44139 USA).In experiments to confirm the binding of mCD137 and CRDs to mCD137L on gel, purified proteins of mE,mE4,mE3,mE2,mCD28 and mL (mCD137L-mFc) in loading buffer with 2-ME were boiled for 5 minutes, or mE-hFc and mE3-hFc in loading buffer without 2-ME and without being boiled, then separated on a 10% SDS polyacrylamide gel and blotted onto a nitrocellulose membrane. The membrane was incubated with 1 µg/ml mCD137L-mFc, after blocking, goat anti-mouse IgG was added for detection.

## Results

### Expression and quantification of fusion protein

We constructed three different vectors with human and mouse IgG-Fc and transfected COS-7 cells to express various truncate fusion proteins which were then used in binding assays. According to a quantitative ELISA, the concentrations of expressed proteins in the supernatants of transfected COS-7cells were around 0.5 µg/ml, and the proteins were >80% pure after rProtein A affinity chromatography. The expected monomeric molecular weights, including IgG-Fc, ranged from 32 kD to 48 kD.

### Mouse CD137 Ligand mainly binds to CRDII of the mouse CD137 extracellular region

**Figure 1:**
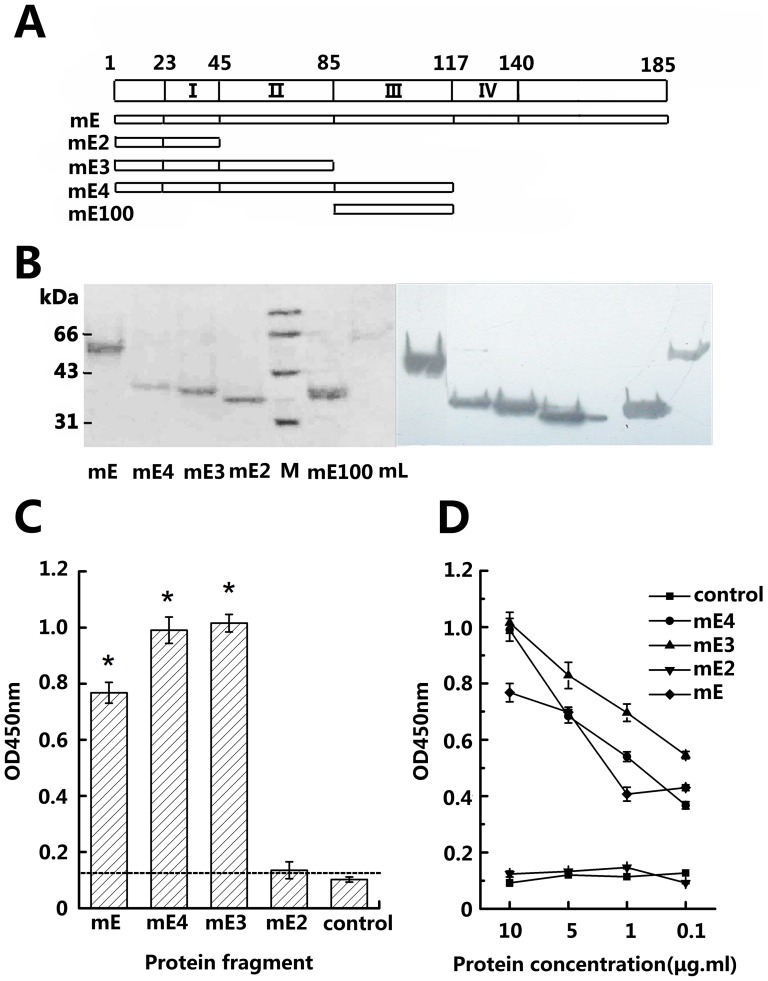
Mapping of mCD137 CRDs binding to mCD137L by ELISA assay. (A) Designs of recombinant murine CD137 proteins. Schematic representation of the entire extracellular region of CD137 is shown on top with the amino acid residue numbers noted, including its leader (1–23 aa). mE, mE4, mE3 and mE2 represent the expressed mCD137 segments covering different CRDs (I, II, III, IV). (B) Identification of purified Fc-fusion proteins expressed with pCDH (mE, mE4, mE3 and mE2) and pCDM-L (mE100 and mCD137L, i.e.mL) in reducing gel: left panel, SDS-PAGE gel with Ponceau S staining; right panel, Western blotting (ECL). (C) Binding of mCD137L to various recombinant mE or subdomains by ELISA assay. Affinity-purified mCD137L (1 µg/ml) was coated on plastic, followed by adding expressed supernatants of mCD137 fragments and subsequently detected by HRP-goat-anti-human IgG. (D) A quantitative binding ELISA in which mCD137L was coated with 1 µg/ml; after blocking with milk, four purified mCD137 protein fragments (mE,mE4,mE3,mE2 ) were added with decreased concentrations 10 µg/ml, 5 µg/ml, 1 µg/ml, 0.1 µg/ml and detected by HRP-goat-anti-human IgG. The binding experiment was repeated 3 times with different batch supernatants. Control was culture supernatant from cells that had not been transfected. *P/N>2.1.

CRDs were constructed as a basic structural unit for the mapping of CD137L binding. Mouse CD137 (referred to as mCD137) has four CRDs among which the structure of CRDII and CRDIII is typical [35]. We designed mE2,mE3,mE4 and mE which cover CRDI, CRDI+II, CRDI+II+III and the full-length mouse CD137 extracellular domain, respectively (Fig. 1A); mE100 covers CRDIII only. Fig. 1B shows the expression of mE,mE2,mE3,mE4,mE100 and mL (mCD137L-mFc) according to SDS-PAGE gel staining and Western blotting. The bands show the proteins have larger molecular weights than expected because the fusion proteins include not only the hinge-like region and Fc fragment but also protein glycosylation. In a pilot assay to measure binding of mCD137L to CD137,wells in an ELISA plate were coated with purified mCD137L and supernatants from mE,mE2,mE3 and mE4 transfected COS-7 cells were added. ELISA assays showed that mE,mE3 and mE4 bound to mCD137L while mE2 (CRDI) did not, and that mE3(CRDI+II) had the strongest binding. Fig. 1C shows data from a representative ELISA assay.

To further validate the binding region, serially decreasing concentrations (10 µg/ml, 5 µg/ml,1 µg/ml,0.1 µg/ml) of purified mE, mE2,mE3,mE4 were added into wells coated with mCD137L. ELISA assays demonstrated that only protein fragments which covered CRDII had a concentration-dependent binding to mCD137L (Fig. 1D).

As the next step, the wells of an ELISA plate were coated with mE100 (CRDIII only) and mE2 (CRDI) after which mCD137L was added to investigate whether CRDIII or CRDI can independently bind to mCD137L.The 450 nm readings for mE100, mE2 and negative control were 0.165, 0.133 and 0.128 respectively, indicating that neither mE100 (CRDIII) nor mE2 (CRDI) can bind to mCD137L. The results were the same when the proteins were coated at 5 µg/ml, further confirming that CRDII is the main region for binding of mCD137L (Table 1).

**Table 1:** Comparison of CD137 core CRDs binding to CD137L between mouse and human.

rCD137 segments,m/h	Covered CRDs	Extent of amino acid,m/h	Size of predicted protein,kD	Binding to ligand,m/h
E	I II III IV STP	1–185/1–186	48	+/+
E4	I II III	1–117/1–118	41	+/+
E3	I II	1–85/1–86	37	+/−
E2	I	1–45/1–46	33	−/−
E100/E3003	III	86–117/87–118	34	−/−

Note: rCD137, Recombinant CD137; +,binding; −,no binding; STP, hinge-like region; m/h, mouse/human.

### Human CD137 Ligand mainly binds to CRDIII in the human CD137 extracellular region

**Figure 2:**
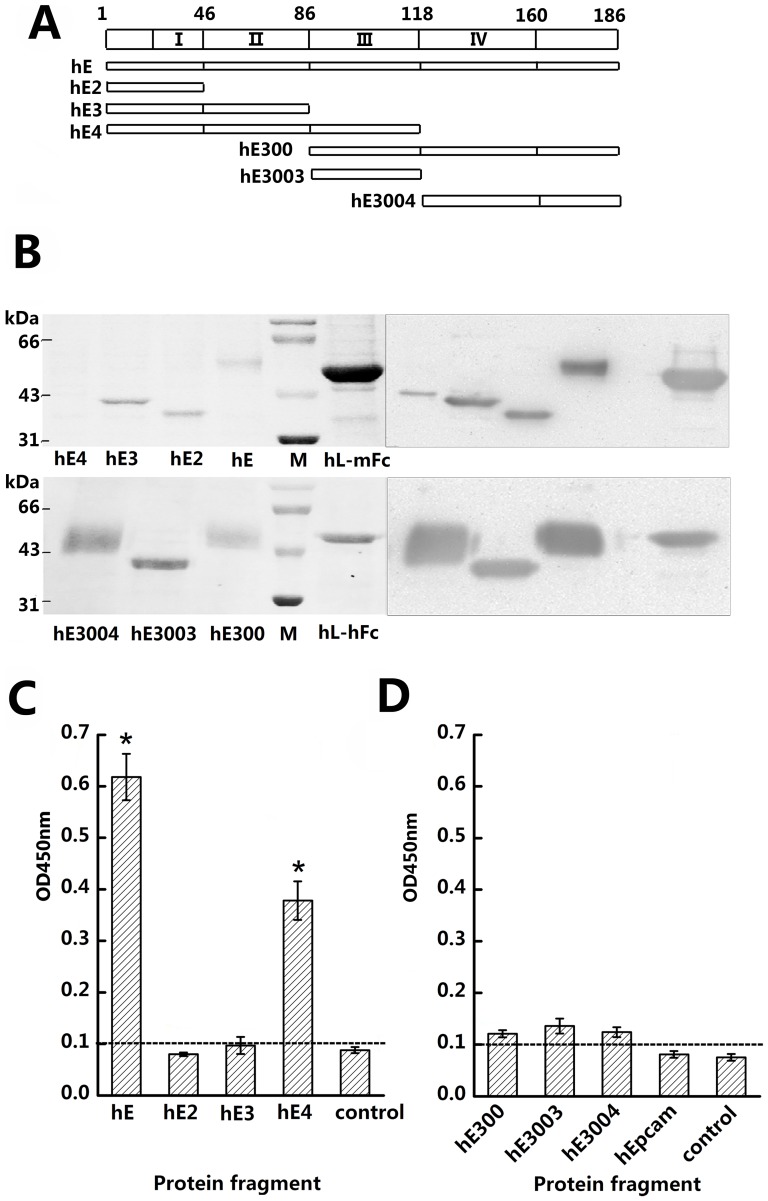
Mapping of hCD137 CRDs binding to hCD137L by ELISA assay. (A) Diagram of hCD137 entire extracellular region and hE, hE2, hE3, hE4, hE300, hE3003 and hE3004 represent the expressed hCD137 segments covering different CD137 CRDs used in this study. (B) Identification of purified expressed series of Fc-fusion proteins with Ponceau S staining of SDS-PAGE gel (left top panel) and ECL (right panel). hE, hE2, hE3, hE4 and hCD137L-hFc (hL-hFc ) were expressed by pCDH and pCDH-L respectively.hE300, hE3003, hE3004 and hCD137L-mFc ( hL-mFc ) were expressed by pCDM-L. (C) Binding of hCD137L to various recombinant hE and subdomains by ELISA assay. Affinity-purified hCD137L-mFc (1 µg/ml) was coated on plastic, followed by addition various hCD137 fragments expressed supernatant, then detected by HRP-goat-anti-human IgG. (D) hCD137L-hFc (1 µg/ml) was coated, followed by addition hE300, hE3003 and hE3004 fragments expressed supernatant and also an unrelated epithelial cell adhesion molecule (Epcam) protein with mFc, the binding detected by HRP-goat-anti-mouse IgG., Similar results were obtained in all of 3 individual experiments. *P/N>2.1.

The amino acids of human and mouse CD137 have 73.5% expected similarity and 59.6% identity [2]. The human CD137 extracellular region contains 186 amino acids which is one more than that of mice and the distribution and structure of the CRDs from the two species is very similar with their CRDI and CRDIV regions both containing only four cysteines that cannot form the typical CRD structure while their CRDII and CRDIII both contain six cysteines. Glycosylation sites are located between CRDIV and hinge-like region in both species [36]. Therefore,hE2,hE3,hE4 and hE (human CD137 extracellular region) were designed to cover CRDI, CRDI+II, CRDI+II+III and the full-length CD137 extracellular domain as in the experiments with mouse CRDs. hE300, hE3003 and hE3004 were designed to cover CRDIII+CRDIV, single CRDIII, and single CRDIV(Fig. 2 A,B). ELISA wells were coated with purified hCD137L after which supernatants of hE, hE2, hE3, hE4 expressed cells were added. The hE and hE4 supernatants were positive while the hE2 and hE3 supernatants were negative, indicating that the CRDI+CRDII constructs cannot bind to hCD137L, while CRDI+CRDII+CRDIII can (Fig. 2C).

**Figure 3:**
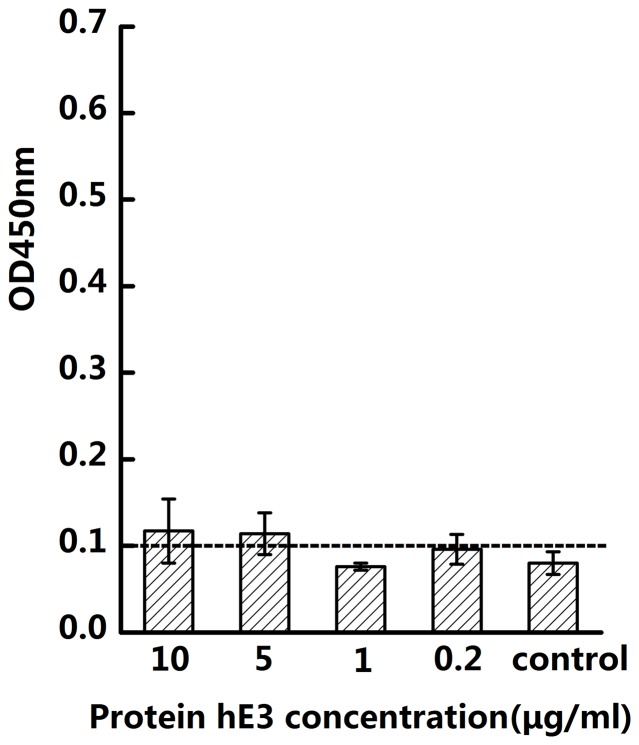
Binding experiment of hE3 to hCD137L by ELISA assay. hE3, which is hIgG1-Fc fusion protein expressed by pCDH, containing hCD137 CRD I+II. hCD137L-mFc was coated at 1 µg/ml and a series of decreasing concentration of hE3, 10 µg/ml, 5 µg/ml, 1 µg/ml and 0.2 µg/ml were added, followed by detecting with HRP-goat-anti-human IgG. The experiment was repeated 3 times with different batch supernatants.

In order to validate whether hE3 can bind to the ligand, purified hE3 protein at different concentrations (10 µg/ml, 5 µg/ml, 1 µg/ml, 0.2 µg/ml), instead of supernatants, were added to ELISA wells coated with hCD137L. No binding was observed (Fig. 3). ELISA assays were also performed with another series of CD137 fragments—hE3003 (separate CRDIII), hE300(separate CRDIII+CRDIV) and hE3004 (separate CRDIV). The results were also negative (Fig. 2D). We conclude that CRDIII is the main binding region for human CD137L, while CRDI+CRDII may be needed to assist CRDIII in contact with human CD137L (Table 1). CRDI+CRDII may less involved in contact also in view of data showing that a CRDII specific anti-human CD137 mAb (4B4-1),which binds to hE3 but not to hE4, cannot interfere with the binding between the ligand and its receptor in the blocking experiments.

### Cross-binding of human CD137 Ligand to mouse CD137

**Figure 4:**
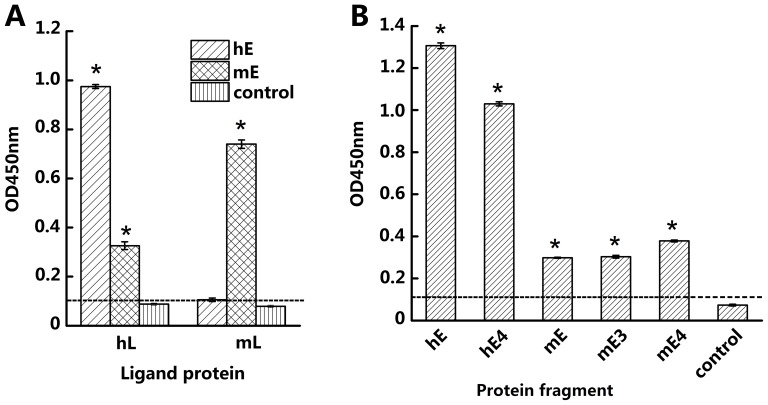
Cross binding of CD137L to CD137 between human and mouse species by ELISA assay. (A) Human or mouse CD137L-mFc (hL/mL) was coated (1 µg/ml) on an ELISA plate. After blocking with milk, supernatant containing the entire human or mouse CD137 extracellular domain (hE, mE) with human IgG tail was added and followed by HRP-goat-anti-human IgG for detection. (B) hL-mFc was coated to an ELISA plate and supernatants containing hE, hE4, mE, mE3 and mE4 all with h-Fc tail fusion proteins were added. hE and hE4 were used as positive controls and supernatant from cells that had not been transfected was used as a negative control. HRP-goat-anti-human IgG was added for detection. The figure shows representative results from one of three experiments. *P/N>2.1.

**Figure 5:**
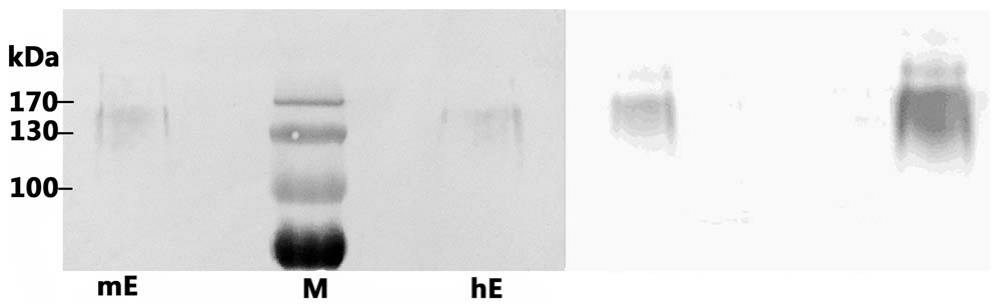
Cross binding of hCD137L to mCD137 via PAGE gel. mCD137 extracellular region (mE) and hCD137 extracellular region (hE) were run on 10% SDS-PAGE gel without 2-ME, then stained by Ponceau S(left panel) and followed by Western blotting with hCD137L-mFc and HRP-goat-anti-mouse IgG for detection (ECL,right panel).

Purified human CD137L (hL) was coated to ELISA wells at 1 µg/ml and expression supernatants of human CD137 (hE) and mouse CD137 (mE) were added. As shown in Fig. 4A, both hE and mE can bind to hL, although the binding by hE is greater. In a parallel experiment, purified mouse CD137L (mL) was used to coat the plastic wells at 1 µg/ml after which hE and mE supernatants were added. As shown in Fig. 4A, mE binds to mL while hE does not. In order to verify the binding of hCD137L to mCD137, ELISA plates wells were coated with hL and expression supernatants of mE3 and mE4 were added. As illustrated in Fig. 4B, both mouse CD137 fragments can bind to hCD137L, but binding affinity of mouse fragments to hL is much lower than that of hE to hL. Furthermore, when a SDS-PAGE-ECL assay was used for binding analysis under non-reduced condition (i.e. without 2-ME), both hE and mE were detected with dimers and the MW was twice that of predicted monomers (data not shown). While the protein amounts of hE and mE were similar on gel (Fig. 5 left panel), the ECL result showed that the hCD137L binding to hCD137 was much stronger than to mCD137 (Fig. 5 right panel). We conclude that hCD137L can bind to mCD137.

### CD137 binding to CD137 Ligand is related to the conformational structure

**Figure 6:**
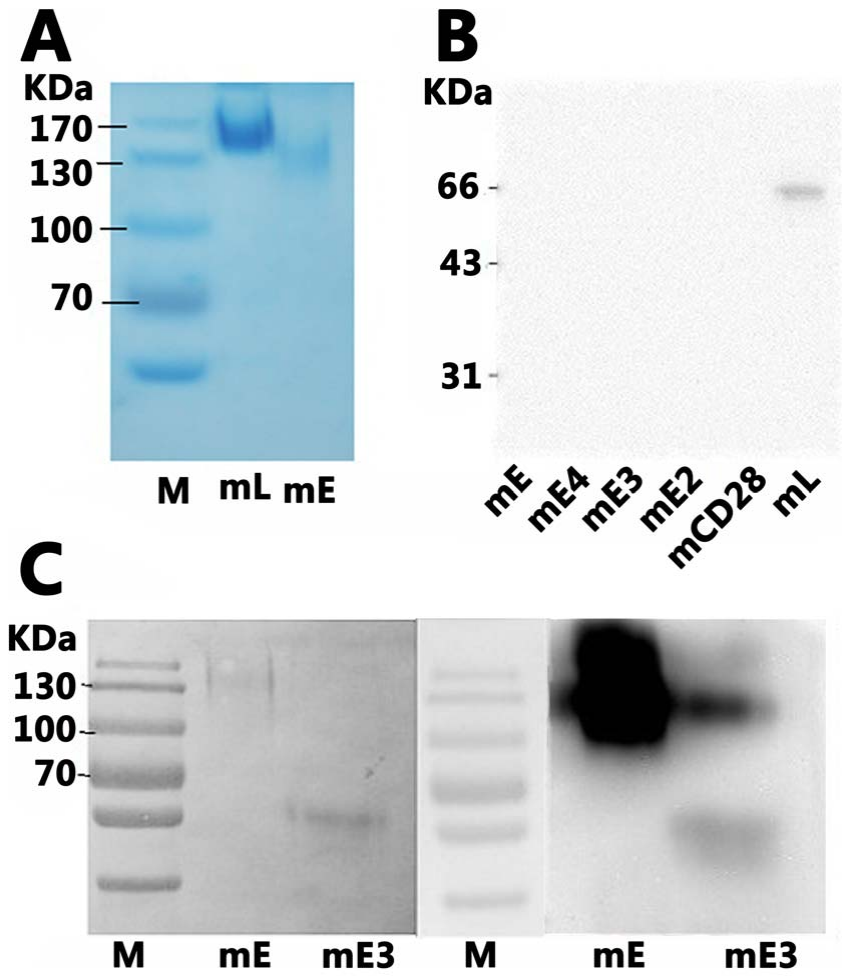
Identification and binding analysis of mCD137 to mCD137L via PAGE gel. (A) Identification of purified mL ( mCD137L-mFc ) and mE ( mCD137-hFc ). Samples were run on a 10%SDS-PAGE gel without 2-ME by Coomassie brilliant blue staining. (B) Western blot analysis of mE,mE4,mE3,mE2 binding to mCD137L. mCD28 and mL were used as negative or positive controls, respectively. Western blot analysis was performed on a 10%SDS-PAGE gel with 2-ME. Bound mL was detected by HRP-goat-anti-mouse IgG. (C) Western blot analysis of mCD137L binding to mCD137 via 10%SDS-PAGE gel in the absence of 2-ME. mE and mE3 run on 10%SDS-PAGE gel, followed by detecting with mL and HRP-goat-anti-mouse IgG in succession. Left panel, Ponceau S staining. Right panel, Western blotting (ECL).

mL-Fc and mE-Fc were separated by SDS-PAGE without 2-ME, and Fig. 6A demonstrates that both the ligand and the receptor exist as dimers. Subsequently, mE, mE2, mE3, mE4, mCD28 (all with hFc tail) and mL (mCD137L–mFc, as a control for anti-mFc detection) were run on SDS-PAGE gel with 2-ME after which the proteins were transferred to a nitrocellulose membrane and incubated with mCD137L (mL-mFc) for ECL. Only mCD137L was detected while the other proteins were all negative (Fig. 6B). However, when mE and mE3 were run on SDS-PAGE without 2-ME, followed by incubation with mCD137L for the ECL, both mE and mE3 bound to mCD137L (Fig. 6C). These results indicate that the binding of CD137 to its ligand involved to intrachain disulfide bonds which are necessary for maintaining the CRD's intramolecular structural stability [2].

### Anti-CD137mAbs specific to CRDs that predominantly bind to CD137 Ligand with high blocking efficiency

**Figure 7:**
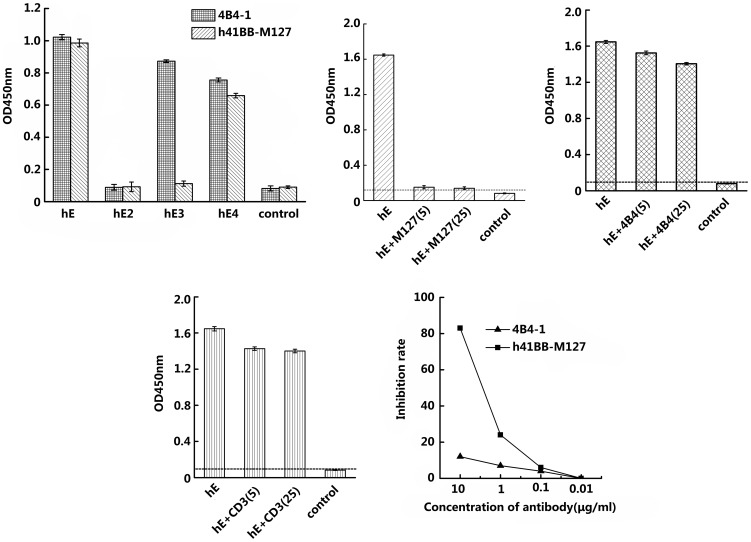
Blocking the binding of hCD137L to hCD137 by anti-human CD137 mAbs 4B4-1 and h41BB-M127. Panel A shows the binding of the two mAbs to hE, hE2, hE3, hE4 and control. Panels B, C and D shows an experiment where human CD137L-mFc (hL) was coated (1 µg/ml) on an ELISA plate, blocked with milk and followed by adding hE(1 µg/ml) which had been preincubated with h41BB-M127 (B), 4B4-1 (C) and an anti-human CD3, OKT3 (D) at a concentration of 5 µg/ml or 25 µg/ml. (E) Inhibition of the binding of hCD137L to hCD137 at a series of concentrations of anti-human CD137mAbs.

Two anti-human CD137 mAbs with different epitopes specificity, clone 4B4-1, which recognizes hCD137 CRDII by binding to hE3 and clone h4-1BB-M127, which recognizes hCD137 CRDIII by binding to hE4 but not to hE3 (Fig. 7A), were tested for their ability to interfere with the binding of hCD137 to hCD137L. At concentrations of 5 µg/ml and 25 µg/ml, h4-1BB-M127 fully blocked the binding (Fig. 7B), while the 4B4-1 mAb did not interfere with the binding (Fig. 7C) any more than the negative OKT3 control (Fig. 7D). In order to determine the efficiency of the mAbs to inhibit this binding, we varied their concentrations in the mixture from 10 µg/ml to 0.01 µg/ml and found that h4-1BB-M127 had an inhibitory effect at 1 µg/ml, which increased (>80%) when it was applied at 10 µg/ml. In contrast, even at 10 µg/ml, the 4B4-1 mAb only showed around 10% blocking effect (Fig. 7D). This finding indicates that CRDIII is involved in the contact of human CD137 with human CD137L.

**Figure 8:**
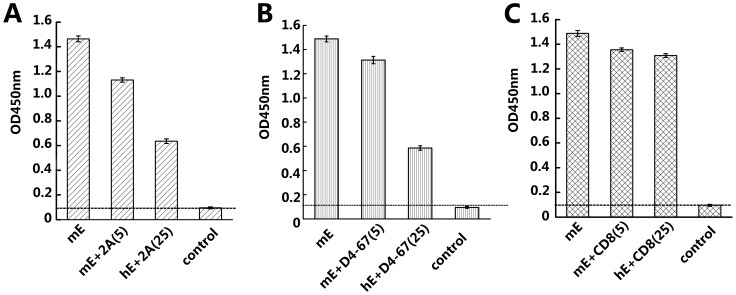
Blocking of the binding of mCD137L to mCD137 by anti-mouse CD137 mAbs. The binding ability of mCD137L to mCD137 under rat anti-mouse CD137mAbs interference was measured using an ELISA assay. Mouse CD137L-mFc (mL) was coated (1 µg/ml) on an ELISA plate and blocked with milk which was followed by adding mouse CD137-hFc extracellular domain (mE, 1 µg/ml) that had been preincubated with rat anti-mouse CD137mAb from clone 2A (A), clone D4-67 (B) or rat anti-mouse CD8 (C); the mAbs were used at a concentration of either 5 µg/ml or 25 µg/ml, and mouse anti-human-Fc-HRP was used for detection. Representative results are shown from one of three similar experiments.

In similar experiments, two rat anti-mouse CD137 mAbs, D4-67 and 2A, were tested for their ability to block the binding of mCD137L to mCD137. The mAbs are specific for CDRIII+CDRIV or a hinge-like region immediately adjacent to the transmembrane domain, respectively (data not shown). They modestly inhibited the mCD137-mCD137L binding at a concentration of 5 µg/ml and inhibited ∼50% of binding when the concentration reached 25 µg/ml (Fig. 8A and B). The experiments support the conclusion that there is a discrepancy with respect to the location of the CD137-CD137L interaction between human and mouse.

## Discussion

We have mapped the CRDs for the CD137L binding to CD137 in both mouse and human. A mouse CD137 extracellular fragment contained CRDI+II bound to mouse CD137L equally well as the whole mouse CD137 extracellular domain, while the CRDI alone could not bind to mCD137L. In contrast, human CRDI+CRDII could not bind to human CD137L while CRDI+CRDII+CDDIII could. We conclude that the binding between CD137 and its ligand primarily involves CRDII in the mouse and CRDIII in human. The possibility that CRDII plays a role in the binding of human CD137 to its ligand seems unlikely also in view of our finding that the anti-human CD137 mAb 4B4-1 which binds to CRDII, could not block the hCD137L-hCD137 binding, while the anti-human CD137 mAb h4-1BB-M127, which binds to CRDIII, blocked this binding. When studying other TNF family numbers such as TNF1/TNFR1 and TNF10/TNFR10B crystallographic complexes, specific patches were detected on the receptor side, involving the second and third CRDs (CRD2 and CRD3) and appear to play a major role in modulating the binding affinity and specificity [24]–[25], [37]. The literatures in reference to CD137 were anticipated to bind their ligand through the conservation of the CRDII and CRDIII [36]. Recently, It has been reported that CRD1, also named pre-ligand-binding assembly domain (PLAD), is physically distinct from the domain that forms the major contact with ligand, and there is increasing evidence for PLAD-mediated receptor association [38].

Cross-binding of CD137L to CD137 was observed between human and mouse. The CD137-CD137L cross-binding showed the following characteristics: (i) Human CD137L can bind to mouse CD137, but mouse CD137L cannot bind to human CD137. The combining affinity of hCD137L to mouse CD137 is about 30% of that of human CD137. (ii) hCD137L cross-binding to mCD137 is mapped to mouse CD137 CRDII or CRDII+CRDIII. Although some TNFL/TNFR interactions are mutually exclusive, cross-interactions have been reported in a majority of cases [39].

Typical TNFR family members possess four CRDs that can be distinguished on the basis of primary sequence characteristics. Direct structural analysis of several TNFL:TNFR family complexes demonstrates that the majority of the residues contacting the cognate ligands are contributed by CRD2 and CRD3, with CRD1 and CRD4 making few contacts [37]. The contact regions between ligands and receptors are diverse in the third group of TNF family members. For example, both human OX40 and CD40 receptors use CRD1,CRD2, and CRD3 regions to bind their respective ligands. The binding interface lacks a single “hot spot” in OX40 [30], whereas in the CD40-CD154 receptor-ligand binding is concentrated to two areas [29]. Our data indicate that only one CRD is predominantly responsible for the CD137L to receptor binding both in human and mouse.

It is unclear what causes the different CRDs in humans and mice to interact with their respective CD137L. We first sequentially analyzed the CRDII and CRDIII for human and mouse CD137 and found that the constitutive amino acid length of the CD137 extracellular region was almost identical (Table 1). Alignment of the human and murine CD137 amino acid sequences has indicated 60% identity between the two species and analysis of the aligned amino acid sequence of CRDII and CRDIII showed 65% and 71% identity, respectively. Furthermore, the numbers of hydrophilic and hydrophobic amino acids of human and mouse CD137 are similar (Table S2), which are 12/15 in CRDII and 10/10 in CRDIII of hydrophilic amino acid; 19/19 in CRDII and 12/11 in CRDIII of hydrophobic amino acid in reference to their sequence [2]. The cross-binding of hCD137L to mCD137 may be related to the similarity of the respective CRD's, although mutational analysis of the CRDII and CRDIII will be needed to obtain more detailed information on the ligand-CRD's interaction. There is only 25%–30% amino acid similarity between TNF-like ligands which is largely confined to internal aromatic residues of assembled constructs [40], and there is only 36% amino acid indentity in the CD137 proteins of mouse versus human origin [2], [31]which may contribute to the different ligand-receptor bindings. Crystal structure of the CD137L-CD137 complex and site-directed mutagenesis experiments are needed to further indentify the important CRDII and CRDIII residues for ligand-receptor binding and gain insight whether any additional CRDs are also involved. There is much interest to develop novel therapies by using agonist and antagonist mAbs to CD137 [41]–[42], and exact mapping of the CD137-CD137L binding should aid future research on CD137 and help the production of antibodies that are either agonistic or antagonistic and improve in vivo efficacy versus toxicity.

## Supporting Information

Table S1 The primers used in RT-PCR for extracellular domains of CD137(E) and CD137L(L).
(DOC)

Table S2 Alignment of the human and murine CRDs' amino acid sequences.
(DOC)
