# Supplementary material for: Human and Mouse CD137 Have Predominantly Different Binding CRDs to Their Respective Ligands
Source: PLoS One. 2014 Jan 21;9(1):e86337. doi: 10.1371/journal.pone.0086337 (PMC3897701; doi:10.1371/journal.pone.0086337)
Supplement: Table S1 — The primers used in RT-PCR for extracellular domains of CD137(E) and CD137L(L). (DOC) [file pone.0086337.s001.doc]

Table S1. The primers used in RT-PCR for extracellular domains of CD137(E) and CD137L(L)

| Target gene | Forward primer(5’-3’) | Reverse primer(5’-3’) |
| --- | --- | --- |
| mE | cgcaagcttcgccatgggaaacaactgttacaacgt | ccgctacgtaggatccctgcaaggagtgccctcct |
| mE2 | cgcaagcttcgccatgggaaacaactgttacaacgt | cgcggatcccttgcagactggattgtattttctgca |
| mE3 | cgcaagcttcgccatgggaaacaactgttacaacgt | aaaggatccctcacactccgcgttgtgggtagagga |
| mE4 | cgcaagcttcgccatgggaaacaactgttacaacgt | aaaggatcctttgcaaccctgcttcgttagctcctg |
| mE100 | caccggtgcagagtgcattgaaggattccattgctt | aaaggatcctttgcaaccctgcttcgttagctcctg |
| mL | attaccggtgcaaccgagcctcggccagcg | tttggatccttcccatgggttgtcgggtt |
| hE | tttaagcttcatcatgggaaacagctgttacaac | tttagatctctgcggagagtgtcctggctct |
| hE2 | tttaagcttcatcatgggaaacagctgttacaac | cgcagatctactgcaaatctgattcctgttattatc |
| hE3 | tttaagcttcatcatgggaaacagctgttacaac | aaaagatctgtcacactctgcattgctggtggagga |
| hE4 | tttaagcttcatcatgggaaacagctgttacaac | cgcagatctgtctttacaacctttttttgtcag |
| hE300 | agaaccggtgcagactgcactccagggtttc | tttagatctctgcggagagtgtcctggctct |
| hE3003 | agaaccggtgcagactgcactccagggtttc | cgcagatctgtctttacaacctttttttgtcag |
| hE3004 | tttaccggtgcagactgttgctttgggaca | tttagatctctgcggagagtgtcctggctct |
| hL | attaccggtgcagcctgcccctgggccgtg | attggatccttccgacctcggtgaagggag |
