# Supplementary material for: Human and Mouse CD137 Have Predominantly Different Binding CRDs to Their Respective Ligands
Source: PLoS One. 2014 Jan 21;9(1):e86337. doi: 10.1371/journal.pone.0086337 (PMC3897701; doi:10.1371/journal.pone.0086337)
Supplement: Table S2 — Alignment of the human and murine CRDs' amino acid sequences. (DOC) [file pone.0086337.s002.doc]

Table S2. Alignment of the human and murine CRDs' amino acid sequences

| CRD | Species | Whole sequence | Hydrophilic number | Hydrophobic number |
| --- | --- | --- | --- | --- |
| II | human | PCPPNSFSSAGGQRT CDICRQCKGVFRTRKECSSTSNAEC | 12 | 19 |
|  | mouse | SCPPSTFSSIGGQPN CNICRVCAGYFRFKKFCSSTHNAEC | 15 | 19 |
| III | human | DCTPGFHCLGAG CSMCEODCKQGQELTKKGCK | 10 | 12 |
|  | mouse | ECIEGFHCLGPQ CTRCEKDCRPGQELTKQGCK | 10 | 11 |
